# Silencing of Profilin-1 suppresses cell adhesion and tumor growth via predicted alterations in integrin and Ca^2+^ signaling in T24M-based bladder cancer models

**DOI:** 10.18632/oncotarget.12218

**Published:** 2016-09-23

**Authors:** Maria Frantzi, Zoi Klimou, Manousos Makridakis, Jerome Zoidakis, Agnieszka Latosinska, Daniel M. Borràs, Bart Janssen, Ioanna Giannopoulou, Vasiliki Lygirou, Andreas C. Lazaris, Nicholas P. Anagnou, Harald Mischak, Maria G. Roubelakis, Antonia Vlahou

**Affiliations:** ^1^ Proteomics Laboratory, Biotechnology Division, Biomedical Research Foundation of the Academy of Athens, Athens, Greece; ^2^ Research and Development Department, Mosaiques Diagnostics GmbH, Hannover, Germany; ^3^ Laboratory of Biology, School of Medicine, National and Kapodistrian University of Athens, Athens, Greece; ^4^ Cell and Gene Therapy Laboratory, Biomedical Research Foundation of The Academy of Athens, Athens, Greece; ^5^ Research and Development Department, GenomeScan B.V., Leiden, The Netherlands; ^6^ First Department of Pathology, School of Medicine, National and Kapodistrian University of Athens, Athens, Greece

**Keywords:** Profilin-1, bladder cancer, xenograft, gene silencing, metastasis

## Abstract

Bladder cancer (BC) is the second most common malignancy of the genitourinary system, characterized by the highest recurrence rate of all cancers. Treatment options are limited; thus a thorough understanding of the underlying molecular mechanisms is needed to guide the discovery of novel therapeutic targets. Profilins are actin binding proteins with attributed pleiotropic functions to cytoskeletal remodeling, cell adhesion, motility, even transcriptional regulation, not fully characterized yet. Earlier studies from our laboratory revealed that decreased tissue levels of Profilin-1 (PFN1) are correlated with BC progression to muscle invasive disease. Herein, we describe a comprehensive analysis of PFN1 silencing via shRNA, *in vitro* (by employing T24M cells) and *in vivo* [(with T24M xenografts in non-obese diabetic severe combined immunodeficient mice (NOD/SCID) mice]. A combination of phenotypic and molecular assays, including migration, proliferation, adhesion assays, flow cytometry and total mRNA sequencing, as well as immunohistochemistry for investigation of selected findings in human specimens were applied. A decrease in BC cell adhesion and tumor growth *in vivo* following PFN downregulation are observed, likely associated with the concomitant downregulation of Fibronectin receptor, Endothelin-1, and Actin polymerization. A decrease in the levels of multiple key members of the non-canonical Wnt/Ca^2+^ signaling pathway is also detected following PFN1 suppression, providing the groundwork for future studies, addressing the specific role of PFN1 in Ca^2+^ signaling, particularly in the muscle invasive disease.

## INTRODUCTION

Bladder cancer (BC) constitutes the second most frequent cause of mortality among genitourinary cancers, accounting for approximately 429.000 cases and 165.000 deaths annually worldwide [1]. It is classified into non-muscle invasive (NMIBC; stages pTa, pT1) and muscle invasive BC (MIBC; stages pT2^+^) [1, 2]. Treatment strategies are limited [3, 4] and radical cystectomy is the standard therapeutic option for MIBC [3]. A better understanding of the molecular events associated with BC invasion can lead to the discovery of potential drug targets and this goal is thus of high clinical relevance.

Profilin-1 (PFN1) is a member of the Profilin protein family, including members of 10-15kDa that bind to actin monomers (G-actin) [5]. Although PFN1's function as an inducer of actin polymerization has not been fully elucidated [6], systematic studies indicated that PFN1 promotes actin polymerization through Adenosine diphosphate (ADP) to Adenosine triphosphate (ATP) exchange and ATP-actin localization at the leading edge of actin filaments [7, 8]. PFN1 also presents an intrinsic ability to bind Phosphatidylinositol (4,5)-bisphosphate (PIP2), Phosphatidylinositol (3,4,5)-trisphosphate (PIP3) molecules [9, 10] and other proline rich proteins [11]. As such, PFN1 has been considered to be involved in both cytoskeleton remodelling and the adhesion capacity of the cells [7]. PFNI has been implicated in cancer, including pancreatic [12], gastric [13] and breast [9, 14–16] cancers, with frequently contradicting results; supporting in cases that PFN1 induces tumor establishment or in others, suppressing progression and metastasis [17].

Observations from our lab indicated that PFN1 tissue levels are inversely correlated with BC progression [18]. In brief, tissue microarray analyses revealed a statistically significant decrease of PFN1 expression in the epithelial cells of the invasive BC tumors (pT2^+^), compared to the non-invasive tumors of the high-risk (pT1G3) group [18]. This pattern strongly correlated with poor prognosis and decreased overall survival [18]. *In vitro* blocking studies for PFN1 demonstrated decreased migration ability of the invasive BC T24 cells [18]. In this study, we targeted to further characterize the underlying network of molecular interactions of PFN1 in relation to BC invasion. Towards that end, we performed *in vitro* and *in vivo* silencing experiments in combination to a series of phenotypic and molecular analyses using the metastatic T24M cells. Our results collectively suggest that PFN1 promotes cell migration and adhesion, and these events are likely mediated through interactions involving integrins but also a predicted induction of the non-canonical Wnt/Ca^2+^ signaling pathway.

## RESULTS

### Development of a PFN1-relevant BC xenograft model

**Figure 1:**
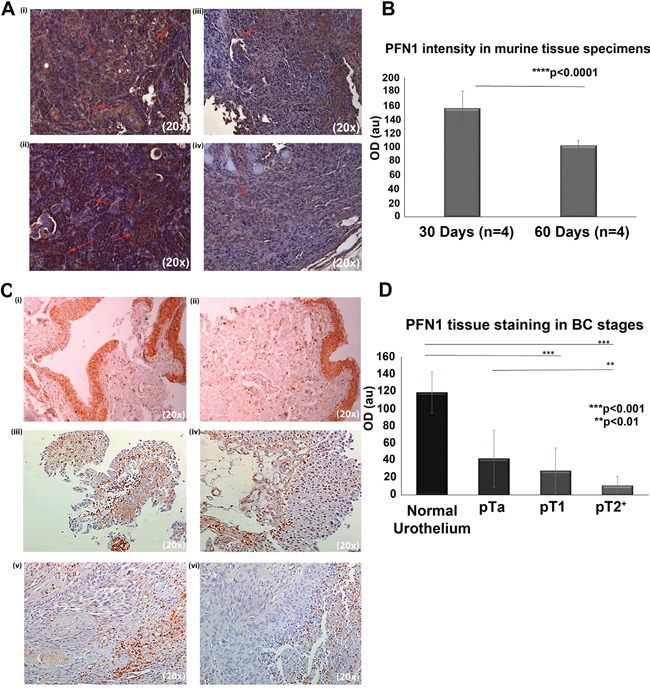
**A.** Representative images from immunohistochemical analysis of PFN1 expression in paraffin-embedded sections derived from T24M tumors in two different time points: 30 days and 60 days after s.c. injection in NOD/SCID mice. (i-ii) Representative images of tumor sections stained for PFN1 at 30 days after s.c. injection. Red arrows indicate PFN1 staining. (iii-iv) Representative images of tumor sections stained for PFN1 at 60 days after s.c. injection. Magnification: 20x. **B.** Relative quantification scores revealing a decrease in PFN1 expression in larger tumors. Values are means ± SD for sections obtained from 4 mice and stained in dublicates (***p< 0.001, Student's t-test). **C.** Representative images of human tissue sections following PFN1 IHC and respective quantification of the PFN1 expression (Image J software). (i-ii) Representative expression of PFN1 in normal adjacent epithelium (n=15), (iii-iv) PFN1 expression in BC tissue from patients with NMIBC, of (iii) pTa stage (n=9) and (iv) pT1 stage (n=8). In pT1 specimens, nuclear staining is also present. (v-vi) Low levels of expression of PFN1 are observed in MIBC cases (pT2^+^, n=7). **D.** Relative quantification scores revealing a decrease in PFN1 expression as tumor progresses. Values are means ±SD. Detailed measurments per case (n=15 slides for normal urothelium, 9 slides for Ta, 8 for T1 and 7 slides for T2+) are provided in Supplementary Table S1 (^**^p< 0.01, ^***^p< 0.001, Student's t-test).

Investigation of PFN1 expression in three BC cell lines showed that its protein levels did not differ signifficantly between the T24 (invasive), T24M (metastatic) and RT4 (non-invasive) cells (data not shown). As such, to monitor PFN1 expression throughout cancer invasion and progression, we generated NOD/SCID xenografts, utilizing the T24M metastatic cell line. PFN1 expression was monitored for 60 days. As shown (Figure 1A and 1B), PFN1 levels decreased with tumor progression (quantified optical absorbance of 155.5±25.0 au at 30 days compared to 102.5±7.4 au at 60 days, *p*<0.0001 Student's t-test). The average tumor size concomitantly increased from 6.9±0.2 mm^3^ (Day 30) to 8.0±2.2 mm^3^ (Days 60). This PFN1 expression pattern is in agreement with our previous observations in human tissue specimens, when comparing non-invasive (T1G3) and invasive T2^+^ cancer [18]. In the present study, analysis all tumor stages was performed (Supplementary Table S1). In particular, 9 pTa, 8 pT1 and 7 pT2^+^ and 15 normal adjacent tissue sections (from human specimens obtained following surgery) were stained for PFN1 (Figure 1C and 1D). Interestingly, cytosolic, as well as nuclear PFN1 localization was observed. Quantification of the IHC intensity revealed a reduction of PFN1 expression as cancer stage progresses. Statistical analysis of the optical density (OD) values supported a significant difference between PFN1 expression levels in normal (adjacent) and cancer urothelial cells regardless of the tumor stage (*p*<0.0001) [Figure 1C and 1D (i)], as well as between the non-invasive pTa, pT1 and the invasive pT2^+^ stages (*p*=0.007). However, no significant difference was observed in-between the NMIBC (pTa and pT1) stages (*p*=0.35).

### Suppression of endogenous PFN1 results in decreased tumor growth in NOD/SCID xenografts

To investigate the impact of PFN1 downregulation, PFN1 expression was suppressed in T24M cells using shRNA lentivirus and respective xenografts were generated (will be referred as T24MshPFN1). The protein expression levels of PFN1 in T24MshPFN1 cells and in those containing the control non-targeting vector (T24MshSCR) were estimated by western blot at 4.3±6.0% and 97.0±18.3%, respectively in comparison to the PFN1 levels in the untransduced T24M cells (set as 100%) [Supplementary Figure 1A (i-ii)]. PFN1 downregulation in T24MshPFN1 cells was statistically significant compared to T24MshSCR (*p*=0.001, Student's t-test) and T24M cells (*p*=0.001, Student's t-test). Similarly, PFN1 transcript levels were estimated at 18±16% and 65±12% for T24MshPFN1 and T24MshSCR cells, respectively (Supplementary Figure 1B). PFN1 mRNA downregulation was statistically significant, compared to T24MshSCR (*p*=0.04, Student's t-test) and T24M cells (*p*=0.008, Student's t-test).

**Figure 2:**
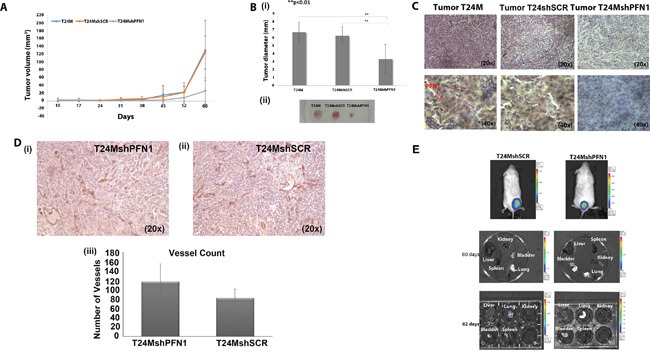
Tumor growth and metastasis analyses in T24M, T24MshSCR and T24MshPFN1 tumor bearing NOD/SCID mice **A.** (i) Tumor volume was significantly smaller in T24MshPFN1 (n=13) compared with T24M (n=16) or T24MshSCR (n=19) tumor bearing animals (^**^p< 0.01, Student's t-test). **B.** Tumor diameters (i) and images (ii) from T24M, T24MshSCR and T24MshPFN1 tumors, 60 days after cell injection in NOD/SCID mice (^**^p< 0.01, Student's t-test). **C.** Immunohistochemical analysis of PFN1 expression in T24M, T24MshSCR and T24MshPFN1 tumor sections. The red arrow indicates the PFN1 staining. Magnifications: 20x, 40x. **D.** Number of tumor vessels, as indicated by CD31 staining, was compared between (i) T24MshPFN1 (n=3) and (ii) T24MshSCR (n=3) tumor bearing animals. Respective statistical analysis (iii) did not show any significant difference. **E.** i) *In vivo* bioluminescence imaging of T24MshSCR LUC (n=4) and T24MshPFN1 LUC (n=4) bearing NOD/SCID mice, after intraperitoneal administration of the substrate of luciferase. Organs were collected: (ii) 60 days and (iii) 82 days after s.c. injections of T24MshSCR LUC and T24MshPFN1 LUC cells. The coloured column indicates the intensity of bioluminescence from lower (purple/blue) to highest (red) intensity.

We further established T24M (n=13), T24MshPFN1 (n=19) and T24MshSCR (n=16) xenografts and monitored them for 60 days. Tumor growth was significantly inhibited in T24MshPFN1 xenografts in comparison to the controls, tumor diameter of 3.3±1.7mm compared to 6.3±1.1 mm (p<0.01, Student's t-test) in T24MshSCR and 6.7±1.4 mm (*p*<0.001, Student's t-test) in T24M tumor bearing mice [Figure 2A and 2B (i-ii)]. Immunohistochemical analysis at T24MshPFN1 tumor sections confirmed the absence of PFN1 protein expression even 60 days after the cell injection (Figure 2C).

To monitor metastases, we employed Xenogen live imaging analysis in xenografts established with cells transduced by the Luciferase virus and the shPFN1 virus (luc-T24MshPFN1) or the shSCR virus (luc-T24MshSCR) (Supplementary Text 1). Since co-transduction of T24M cells with two viruses may affect their transduction efficiency, the employment of un-transduced or transduced with a single virus T24M cells as control, could be misleading. Therefore, we utilized luc-T24MshSCR cells as the only control condition. The results obtained revealed metastasis in lung in T24MshSCR xenografts only (1/4 animals examined). However, no indication of metastasis was detected in the T24MshPFN1 xenografts (n=4) (Figure 2E).

As in some cases, PFN1 has been suggested to impact on angiogenesis [19], we investigated whether the observed decrease in tumor growth or metastasis correlates with a decrease in angiogenesis following PFN1 suppression. Towards that end, immunohistochemical analysis for Platelet endothelial cell adhesion molecule (PECAM-1 also known as CD31) was performed. As shown (Figure 2D -iii) the number of vessels did not differ significantly between T24MshPFN1 and T24MshSCR [116±39 counted vessels for T24MshPFN1; versus 82±19 counted vessels for T24MshSCR; (p=0.28, Student's t-test) Figure 2D (iii)] suggesting that the observed impact is unlikely to involve reduced vessel formation.

### PFN1 suppression impairs adhesion and motility of T24M cells

**Figure 3:**
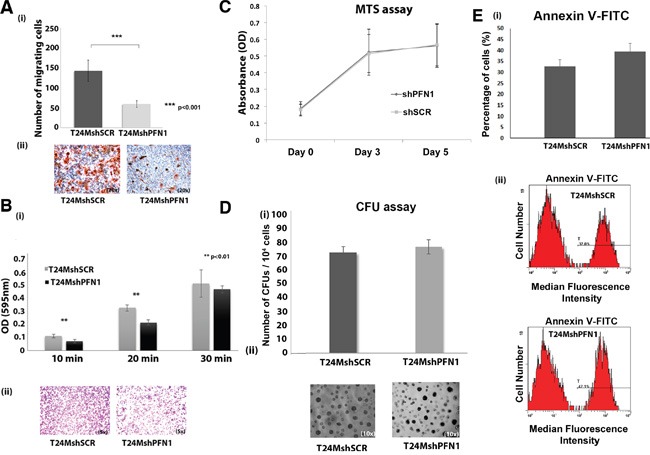
**A.** Downregulation of PFN1 results in decreased motility of T24M cells. (i) Bar graph showing the number of migrating T24MshSCR and T24MshPFN1 cells toward CM derived from T24M cells. Values are means ± SD for two independent experiments (^**^p< 0.01, ^***^p< 0.001, Student's t-test). (ii) Representative images of migrated T24MshSCR and T24MshPFN1 cells. Magnification: 20x. **B.** Downregulation of PFN1 results in reduction of adhesion capacity of T24M cells to fibronectin. (i) Bar graph presenting the average optical absorbance at 595 nm of adherent T24MshSCR and T24MshPFN1 cells to fibronectin for 10, 20 and 30 minutes. Values are means ± SD for three independent experiments (^**^p< 0.01, Student's t-test). (ii) Representative images of adherent T24MshSCR and T24MshPFN1 cells after staining with crystal violet. Magnification: 5x. **C.** Diagram presenting T24MshPFN1 and T24MshSCR cell proliferation rate at three time points (Day 0, Day 3, and Day 5). The optical absorbance given was measured at 595 nm. **D.** (i) Bar graph presenting the mean number of colonies formed by T24MshSCR and T24MshPFN1 cells. (ii) Representative optical images of T24MshSCR and T24MshPFN1 colonies. Magnification: 10x. **E.** Bar graph and representative flow cytometry plots of median fluorescence intensity (MFI) of Annexin-V-FITC staining in T24MshPFN1 cells and T24MshSCR. Values are means ± SD for three independent experiments.

We further investigated the role of PFN1 in migration and adhesion of T24M cells. PFN1 suppression in T24M cells resulted in decreased motility (59±8 migratory cells), compared with T24MshSCR (142±27 migratory cells; *p*<0.001, Student's t-test) when cells were left to migrate toward conditioned media (CM) derived from T24M cells (Figure 3A). There was no statistically significant difference in the migration ability of T24M (191±7) compared to T24MshSCR (142±27) cells (*p*=0.7, Student's *t*-test). This observation was consistent throughout all the *in vitro* experiments. Therefore, we will present the T24MshSCR cells as the only control condition and each comparison described below will refer to the T24MshPFN1 versus T24MshSCR cells.

T24MshPFN1 cells also exhibited statistically significant impaired adhesive properties, when plated on fibronectin for 10 minutes (absorbance at 64%; *p*=0.008 Student's t-test) and 20 mins (66%; *p*=0.003 Student's t-test), when compared with the T24MshSCR cells (absorbance set as 100%) [Figure 3B (i-ii)]. No significant effect on cell adhesion at 30 minutes (absorbance at 91% for T24MshPFN1 cells in comparison to controls; *p*=0.52) [Figure 3B (i-ii)].

### PFN1 suppression does not affect the proliferation, colony formation and apoptotic properties of T24M cells

As shown in the Figure 3C, no significant difference was observed in the proliferation rate of T24MshPFN1 cells after 5 days in culture [Day 0 (0.18±0.04 au, 595 nm), Day 3 (0.52±0.14 au) and Day 5, (0.56±0.13 au], compared with T24MshSCR cells (Day 0 (0.17±0.03au), *p*=0.88, Student's t-test; Day 3 (0.51±0.11 au), *p*=0.93, Student's t-test; Day 5 (0.57±0.13 au), *p*=0.93, Student's t-test). Likewise, no significant alteration in the average number of colonies by T24MshPFN1 (143±93) compared to T24MshSCR cells [(136±89), *p*=0.54, Student's t-test] [(Figure 3D (i-ii)] was observed. In addition, downregulation of PFN1 in T24M cells, exhibited no significant effect in apoptosis, as indicated by Annexin V- fluorescein isothiocyanate staining (FITC) staining [Median Fluorescence Intensity (MFI of 95±5], compared to MFI of 83±11 in T24MshSCR cells (*p*=0.36, Student's t-test) (Figure 3E).

### PFN1 promotes actin polymerization and alters the expression of cell surface molecules

**Figure 4:**
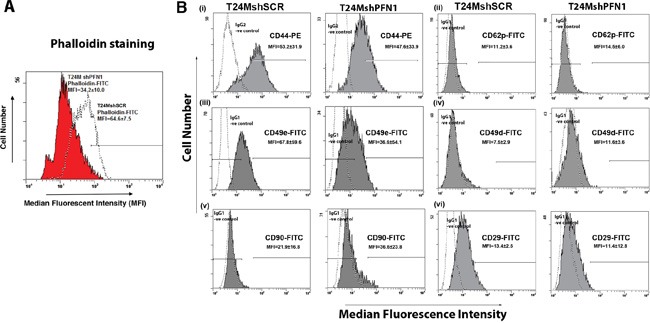
Downregulation of PFN1 results in alterations in actin polymerization and cell surface antigen expression **A.** Representative FACS flow cytometry plot indicating the decreased phalloidin-FITC uptake from T24MshPFN1 cells compared with T24MshSCR cells. **B.** Representative flow cytometry FACS plots for (i) CD44, (ii) CD62P, (iii) CD49e, (iv) CD49d, (v) CD90 and (vi) CD29 expression in T24MshSCR and T24MshPFN1 cells. IgG1/IgG2: Isotype control.

We further investigated the effect of PFN1 downregulation in the actin polymerization by phalloidin-FITC staining (Figure 4A). Decreased expression of PFN1 in T24MshPFN1 cells resulted reduced phalloidin uptake (MFI=50±67), compared to the T24MshSCR cells (MFI=81±23) (Figure 4A).

Following the decreased adhesion properties to fibronectin in the T24MshPFN1 cells, the expression of integrin- and fibronectin-associated receptors was further investigated. For this purpose, T24MshPFN1 and T24MshSCR cells were examined for the expression of Very Late antigen-5 (VLA-5 antigen) Integrin alpha5/beta1 (CD29/CD49e), selectin P/CD62P and CD44 by Fluorescence-based flow cytometry (FACS) analysis (Figure 4B). The CD49e and CD29 antigens were both identified downregulated in T24MshPFN1 compared to T24MshSCR cells (Figure 4B), (MFI for CD49e at 82±7 in T24MshSCR compared to 75±5 in T24MshPFN1 cells). Similarly, CD29 was found decreased in T24MshPFN1 (MFI=39±14), compared to T24MshSCR cells (MFI=51±23). Furthermore, CD44 (MFI=54±16) as well as CD62P (MFI=65±27), exhibited lower expression levels in T24MshPFN1 compared to the T24MshSCR cells (MFI=72±23 and MFI=77±6, respectively).

### Proteome array analysis suggests alteration in the expression levels of tumor-associated secreted factors following PFN1 suppression

**Figure 5:**
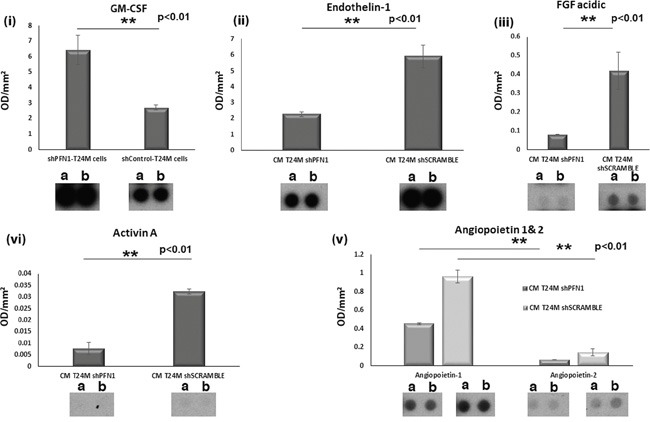
Secretome analysis of T24MshSCR and T24MshPFN1 cells using antibody proteome arrays **A.** Bar graphs and representative spot images presenting the differences at the secretome level of the secreted factors: (i) Granulocyte-macrophage colony-stimulating factor (CSF2) (ii) Endothelin 1, (iii) FGF acidic, (iv) Activin A and (v) Angiopoietin 1 and 2. Each experiment was performed in duplicates.

Proteome array analysis of the secretome of T24MshPFN1 and T24MshSCR cells was performed to investigate the expression of known tumor-associated secreted factors following PFN1 downregulation (Figure 5; Supplementary Figure S2). Endothelin-1 (EDN-1), which enhances cell growth [20], was identified at lower levels (0.38-fold) at the secretome of T24MshPFN1 [Optical Density (OD/mm^2^ of 2.3±0.2 arbitrary units (au)], compared to T24MshSCR cells (OD/mm^2^=5.9±0.7; *p*=0.009, Student's t-test). Additionally, Angiopoietin 1 (ANGPT1) and Angiopoietin 2 (ANGPT2) that regulate migration, adhesion, angiogenesis and tumor growth were observed at decreased levels by 0.46-fold (*p*=0.04, Student's t-test) and 0.43-fold (*p*=0.004, Student's t-test) in T24MshPFN1 (ANGPT1:OD/mm^2^=0.45±0.01 au; ANGPT2:OD/mm^2^=0.06±0.004 au) compared to T24MshSCR cells (ANGPT1:OD/mm^2^=0.96±0.07 au; ANGPT2:OD/mm^2^=0.14±0.04 au). Fibroblast growth factor-1 (FGF-1) and Plasminogen (PLG), both tumorigenic factors, were also decreased in T24MshPFN1 (FGF-1:OD/mm^2^=0.08±0.0004 au and PLG:OD/mm^2^=0.02±0.009 au) compared to the T24MshSCR secretome (FGF-1:OD/mm^2^=0.42±0.09 au) and [PLG:OD/mm^2^=0.04±0.006 au), [0.18-fold *(p*=0.02, Student's t-test) and 0.33-fold (*p*=0.03, Student's t-test), respectively]. Activin A (ACVN1), which is also associated with cancer cell migration and invasion exhibited a 0.24-fold decrease (OD/mm^2^=0.008±0.003 au) in T24MshPFN1 vs T24MshSCR secretome (OD/mm^2^=0.03±0.001 au; *p*=0.009, Student's t-test).

In contrast, the anti-tumor cytokine, Granulocyte-macrophage colony-stimulating factor (GM-CSF) was found at increased levels in the T24MshPFN1 secretome [2.4-fold (*p*<0.01, Student's t-test)], vs T24MshSCR cells. Similarly, Chemokine (C-C Motif) Ligand 2 CCL2 or Monocyte Chemoattractant Protein-1 (MCP-1), a known tumor suppressor, was secreted at higher levels [12.4-fold (*p*<0.007, Student's t-test)] in the T24MshPFN1 secretome (OD/mm^2^=0.58±0.02 au) compared to the secretome of T24MshSCR cells (OD/mm^2^=0.05±0.007 au). Thymidine phosphorylase (TYMP), a key molecule in thymidine cancer therapeutics, was also detected at increased levels [1.56-fold, (*p*<0.01, Student's t-test)] in T24MshPFN1 secretome (OD/mm^2^=0.09±0.005 au), compared with T24MshSCR (OD/mm^2^=0.06±0.005). Other tumor suppressors, such as Dipeptidyl peptidase 4 (DPPIV), Pigment epithelium-derived factor (PEDF) and SerpinB5 were also upregulated in the secretome of T24shPFN1 cells, (30.3, 13.6 and 9.7-fold, respectively).

### Analysis of differentially expressed genes in T24MshPFN1 cells confirms changes in secreted factors

In order to get further insight in the molecular changes that lead to tumor inhibition and in parallel confirm some of the observed proteomic changes, total RNA sequencing of the T24MshPFN1, T24MshSCR and T24M cells was performed. 1.403 differentially expressed genes (fold change >1.5; 1.084 up-regulated and 319 down-regulated) between T24MshPFN1 and T24MshSCR cells were reported. Subsequent comparison was performed between T24MshSCR and T24M cells to investigate any similarities related to lentivirus integration. In total, 21 genes reflecting virus specific-induced changes (fold change>1.5) were identified and excluded from the 1.403 differentially expressed genes. Thus, 1.382 differentially regulated genes were further assessed [1077 up-regulated and 305 down-regulated in T24MshPFN1 vs T24MshSCR]. Out of these, 1.045 exhibited the same regulation trend in both of the following comparisons: (i) T24MshPFN1 versus T24MshSCR and (ii) T24MshPFN1 cells versus T24M. These corresponded to 798 genes being up-regulated and 257 down-regulated in T24MshPFN1 vs controls, out of which 652 were protein coding genes (510 up-regulated and 142 down-regulated) and two micro-RNA sequences (Supplementary Table S2).

**Figure 6:**
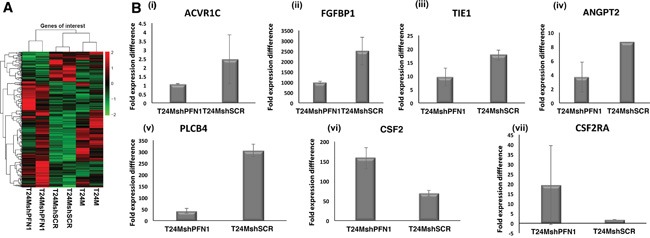
**A.** Heatmap plot generated from RNA sequencing data for T24M, T24MshSCR and T24MshPFN1 cells, analysed in dublicates. **B.** Bar graphs presenting the levels of expression of (i) Activin receptor type-1C (ACVR1), (ii) Fibroblast growth factor-binding protein 1 (FGFBP1), (iii) Tie1 or Tyrosine-protein kinase receptor Tie-1 (ligand of Angiopoietin 1 or Angiopoietin 2), (iv) Angiopoietin 2 (ANGPT2) (v) 1-phosphatidylinositol 4, 5-bisphosphate phosphodiesterase beta 4 (PLCb4), (vi) Granulocyte-macrophage colony-stimulating factor (CSF2) and (vii) Granulocyte-macrophage colony-stimulating factor receptor (CSF2RA) in T24MshPFN1 and T24MshSCR cells.

The results were in agreement with those of the secretome/proteome analysis (Supplementary Table S3): ANGPT2 was down-regulated at the mRNA level, showing a 0.65-fold in the T24MshPFN1 vs T24MshSCR cells (Figure 6). ANGPT1 levels did not pass the 1.5 fold change threshold, nevertheless were slightly decreased (by 0.80). Interestingly, Tyrosine-protein kinase receptor Tie-1 (Tie1), a transmembrane tyrosine-protein kinase that modulates the activity of ANGPT receptors (TEK/TIE2) activity [21] exhibited decreased mRNA levels in T24MshPFN1 (0.60-fold) vs T24MshSCR cells. Moreover, fibroblast-growth factor-1 (FGF1) was downregulated (0.68-fold) in T24MshPFN1 cells, confirming the proteomic results. This also coincided with the decreased mRNA levels of FGF-binding protein-1 (0.60-fold) in T24MshPFN1 versus control, a carrier protein that binds FGFs from the extracellular matrix storage and enhance FGF signaling during angiogenesis and tumor growth [22]. Similarly, Plasminogen was also down-regulated in T24MshPFN1 (0.36-fold) vs T24MshSCR cells. Along these lines, Granulocyte-macrophage colony-stimulating factor-2 (CSF2), as well as its receptor (CSF2RA), were both identified upregulated, in T24MshPFN1 (2.15 and 3.10-fold, respectively) vs T24MshSCR cells. Similarly, CCL2 and TYMPL were also upregulated in T24MshPFN1 vs T24MshSCR cells (CCL2: 2.6-fold; TYMP: 2.1-fold). Although Activin A was not significantly differentially expressed at the mRNA level, Activin receptor type-1C was down-regulated in T24MshPFN1 (0.53-fold) vs T24MshSCR cells. Collectively, mRNA sequencing confirmed the down-regulation of known tumor inducers and up-regulation of tumor suppressors, also supported by the secretome analysis, following PFN1 suppression.

### Functional annotation and pathway analysis predict downregulation of tumor inducing and Wnt/Ca^2+^ pathway following PFN1 suppression

A functional annotation based on Gene Ontology (GO) and pathway analysis was conducted for the differentially expressed mRNAs to investigate their segregation into biological processes (Supplementary Figure S3). Many of the downregulated genes corresponded to proteins involved in cell-cell interactions and cell-extracellular matrix communication [23]. As such, Teneurin-2 has been characterized as a ligand for G protein-coupled receptors promoting adhesion [23]. Here, Teneurin-2 was downregulated in T24M shPFN1 (0.28-fold) vs T24MshSCR cells. Desmocollin-3, a component of desmosome junctions, mediating cell adhesion [24] was down-regulated in T24MshPFN1 (0.65-fold) vs T24MshSCR cells. SH3 and PX domain-containing protein 2A, an adapter protein involved in extracellular matrix degradation and invasiveness of cancer cells via induction of EMT [25] was down-regulated in T24MshPFN1 (0.49-fold) vs T24MshSCR cells. Junctional adhesion Molecule-C (JAM-3) involved in cell adhesion and associated with tumor growth [26] was identified down-regulated in T24MshPFN1 (0.64-fold) vs T24MshSCR cells. In addition, E-Cadherin (CDH1) a tumor suppressor, involved also in cell adhesion, motility and proliferation [27] was identified in higher levels in T24MshPFN1 (3.22-fold) vs T24MshSCR cells. Many differentially expressed mRNAs were found to be involved in inflammatory processes, including chemokines (7%), molecules related to NF-κB signalling (3%) and interferon-gamma signaling (1%) (Supplementary Figure S3). As examples, the C-X-C chemokine receptor type 4 (CXCR4) receptor for the C-X-C motif chemokine 12 (CXCL12) or stromal cell-derived factor 1 (SDF-1) that enhances Mitogen-activated protein kinase (MAPK1/MAPK3) activation [28] was downregulated in T24MshPFN1 cells (0.59-fold). Among the highly up-regulated trancripts in T24MshPFN1 cells (5.18-fold) was Tumor necrosis factor ligand superfamily member-15 (TNFSF15), an angiogenesis inhibitor that suppress tumor growth [29]. Similarly, Tripartite motif containing 31 (TRIM31), reported to suppress colony-forming units (CFU) formation of cancer cells *in vitro* [30], was highly upregulated in T24MshPFN1 cells (5.36-fold).

Interestingly, the majority of the identified molecules are involved in Ca^2+^-driven process (12%) (Supplementary Figure S3), with some additional molecules to be related to G-protein signalling (2%), or to exhibit GTPase (2%) or GTPase regulator (2%) activities. Around 3% of the molecules were classified as phospholipases, protein kinase related proteins and regulators of those. Characteristic examples, include, genes corresponding to phosphatidylinositol-specific phospholipase-C enzymes, such as PLCb4 and PLCb2 (0.19 and 0.51-fold, respectively) most highly down-regulated upon suppression of PFN1; in addition, the Ras-related Rab-3B and Rab-36 proteins were both downregulated (0.55 and 0.61-fold, respectively) in T24MshPFN1 cells.

To further investigate the above findings, *in silico* pathway analysis was conducted. For this purpose, the 652 encoded proteins derived from the 1.045 differentially expressed genes were used as input. After exclusion of redundant entries or those that could not be mapped in the IPA underlying pathways, 645 features segregated into: 280 pathways- with four directly involving PFN1 (Table 1) and 44 identified as statistically significant (*p*<0.05, Fischer's Exact Test) (Supplementary Table S2). This analysis confirmed the functional annotation results, predicting significant changes in: Interferon (*p*=0.004; activated; z=2.236), Chemokine (*p*=0.006; inhibited; z=-0.378), G-Protein Coupled Receptor (*p*=0.008), Phospholipase (*p*=0.008) and NF-κB (*p*=0.02, activated; z=2.111) signaling pathways. Additionally, Axonal Guidance Signaling (*p*=0.02) and the non-canonical planar cell polarity pathway (PCP) (*p*=0.04), directly mapping PFN1 were predicted to be significantly changed following PFN suppression (Table 1).

**Table 1:** Canonical pathways that involve PFN1 and corresponding mapped altered protein coding transcripts, following suppression of PFN1

Ingenuity Canonical Pathways	p-value	Ratio	Molecules
Axonal Guidance Signaling	0.020893	5,16E-02	NFATC4(↑), EPHB6(↑), PLCb2(↓), ADAM8(↑), SRGAP3(↑), GNAO1(↑), PLCb4(↓), SDC2 (↑), EPHA3 (↑), SEMA3B(↑), WNT5B(↓), SEMA6A(↑), PIK3CG(↑), CXCR4(↓), WAS(↑), PFN1(↓), ADAMTS5 (↑), SEMA3C(↑), UNC5B(↑), MMP10(↑), SHH(↑), ADAMTS7(↑)
PCP pathway	0.048978	8,06E-02	RSPO3(↑), PFN1(↓), SDC2(↑), WNT5B(↓), ROR2(↑)
Regulation of Actin-based Motility by Rho	0.149624	5,75E-02	WAS(↑), PFN1(↓), MYLK(↑) RHOU(↑), RND2(↑)
RhoA Signaling	0.54325	3,33E-02	LPAR6(↑), PFN1(↓), MYLK(↑), LPAR3(↓)

The direction of differential expression (↑ or ↓) when comparing T24MshPFN1 vs T24MscPFN1 is shown.

**Figure 7:**
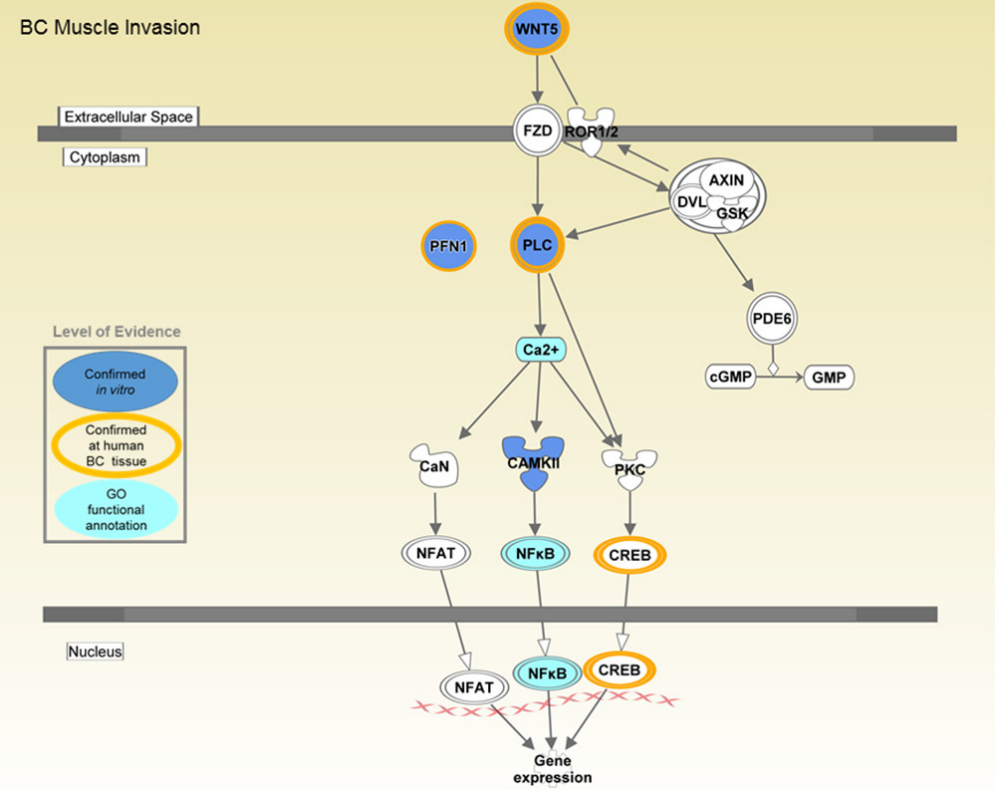
Predicted impact on the non-canonical Wnt/Ca^2+^ signaling following PFN1 suppression Molecules detected at altered levels in T24MshPFN1- versus T24MshSCR transduced cells are marked. An overall decrease in the activity of the pathway is predicted. The pathway was generated by Ingenuity pathway Analysis software (IPA) and further adjusted to depict schematically the main modules that were introduced in this study.

Interestingly, the non-canonical Wnt/Ca^2+^ pathway was predicted to be inhibited/inactivated (z score=-1.00) (Figure 7). Interestingly, with all expressed genes found at decreased levels in in T24MshPFN1 cells versus controls (PLCb4, 0.19 fold change; PLCb2, 0.51 fold change; Wnt5b; 0.46-fold change and Calcium/calmodulin-dependent protein kinase type IV (CAMKIV), 0.65-fold change). As shown in Figure 7, inhibition of the Wnt/Ca^2+^ pathway is predicted to lead to decreased CREB1 and NFkB levels and suppress gene expression mediated via these trascription factors. These data suggest an implication of PFN1 to Wnt/Ca^2+^ pathway integrating the integrin-cytoskeleton axis and PFN1 interaction with PIP2.

### Decrease of Wnt5b, PLC4b and CREB1 in MIBC human tissue

**Figure 8:**
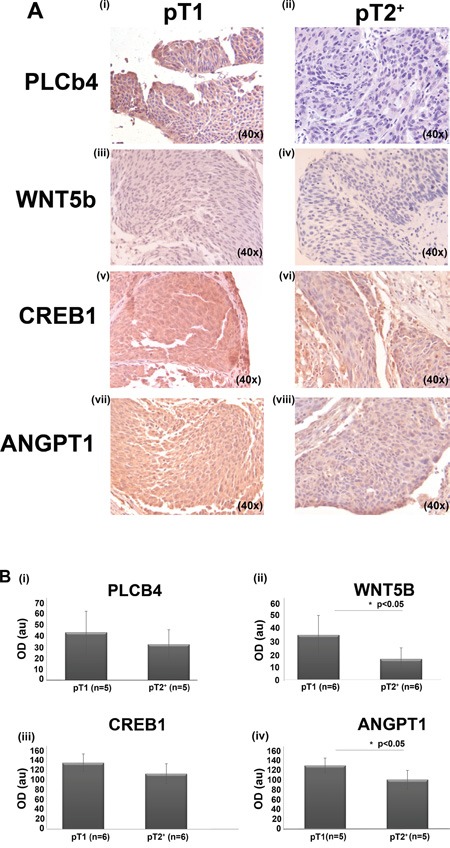
**A.** Representative images of human tissue sections following IHC for PLCB4 (**i-ii;** n=5 for T1; n=5 for T2+), Wnt5b (**iii-iv**; n=6 for T1 and n=6 for T2+), CREB1 (**v-vi**; n=6 for T1; n=6 for T2+) and ANGPT1 (**vii-viii**; n=5 for T1 and n=6 for T2+). **B**. Quantification results (Image J software). Values are means ±S.D. Detailed measurments are included in the Supplementary Table S1 (^*^p< 0.05, Student's t-test).

Considering the following criteria: a) involvement of a protein in altered pathways following PFN1 down-regulation based on the functional annotation/pathway analysis results; b) the differential expression/excretion based on the secretome/proteome analysis and the gene expression data and c) availability of the antibodies; immunohistochemical analysis of bladder cancer tissue sections was further performed for Wnt5b, PLCb4, cAMP response element-binding protein1 (CREB1) and ANGPT1. The selection of these factors was made to increase reliability of the aforementioned observed *in vitro* and *in vivo* changes and pathway analysis predictions. Sections from NMIBC (pT1) and MIBC (pT2+) cases were used. All factors were found to be expressed in the BC tissue specimens (Figure 8A and 8B). Interestingly, quantification of the staining intensity suggested that similar to PFN1 (Figure 1D), the levels of all proteins showed at least a slight decrease in pT2+ versus pT1 BC. This change reached statistical significance in the case of Wnt5b (*p*=0.03) and ANGPT1 (*p*=0.02) in the small set of tested samples.

Correlation of each factor to the Profilin-1 levels per individual sample did not reach statistical significance for Wnt5b, PLCb4, ANGPT1 with the exception of CREB1 which suprisingly showed a strong inverse correlation (p=0.005; ρ=-0.943, Spearman's Rho) to the PFN1 levels in the pT2^+^ samples.

## DISCUSSION

Urothelial tumors arise and evolve through divergent phenotypic pathways [31]. As a result, the response to the already limited treatment schemes is not always achieved [31]. A need for a thorough understanding of the underlying molecular processes is prevailing [32]. We recently identified PFN1 as a tissue biomarker inversely correlated with BC invasion [18]. Previous studies reported that PFN1 has significant role in the promotion of developmental events via Wnt signaling [33], as well as the induction of neuronal cell migration [34]. However, in carcinogenesis, contradictory observations have been reported [12, 13], as PFN1 suppressed tumorigenicity in pancreatic cancer [12, 13], while promoted gastric cancer progression [13]. Studies in breast cancer [9, 14–16], occasionally suggested PFN1 promoting tumor establishment or suppressing metastasis [17]. Collectively, accumulating evidence, support a highly context specific role of PFN1 in carcinogenesis [35] with its molecular interactions not yet been adequately characterized.

In our previous study *in vitro* blocking experiments in T24 cells by the use of neutralizing antibodies resulted in impaired cell migration and actin polymerization [18].

Along the same lines, herein, we show that suppression of PFN1 inhibits T24M cell migration and adhesion *in vitro* and tumor establishment *in vivo*. IHC analyses showed a similar PFN1 expression pattern in human BC and mouse xenograft tissues, with PFN1 levels decreasing with tumor progression. This is in agreement with a study in breast cancer [17], supporting an emerging role of PFN1 in the early tumorigenesis. Our studies further suggested that suppression of PFN1 in T24M cells results in impaired migration and adhesion properties, likely by blocking actin polymerization [35, 36] and suppressing (at least) the VLA-5 antigen, a receptor for fibronectin [37]. Endothelin-1, which has been shown to induce VLA-5 expression [20], was found at decreased secreted levels upon PFN1 suppression. Endothelin-1 has been reported previously to enhance BC metastasis in the lung in mouse BC xenografts [38]. In parallel, cell adhesion to fibronectin may have also been impaired via alterations in the CD44 signaling as suggested by the decreased levels of selectin P/CD62P and CD44 following PFN1 suppression. Thus, we hypothesize that PFN1 suppression has a negative impact on a cascade of events involving ligand binding to integrins, integrin clustering and recruitment of actin filaments, which collectively may contribute to the observed phenotype of impaired adhesion and migration.

Tumor migration may be further suppressed by increased secretion of known tumor suppressor factors following PFN1 suppression. Based on our data, these may include PEDF, earlier reported to inhibit cancer metastasis by down-regulating fibronectin [39]; increased levels of DPPIV and E-cadherin earlier correlated with growth inhibition and apoptosis in melanoma and ovarian cells [40, 41]. GM- CSF (CSF2) and its receptor, that were found at increased levels in the secretome and transcriptome following PFN1 downregulation, is implicated in inducing granulocytes and monocytes and defensive immune response [42]. Along these lines, a vaccine that displayed mouse CSF2 on the surface of BC stem cells induces an anti-tumor immune response to metastatic BC [42]. In addition, combination of GM-CSF vaccination with radiotherapy in patients with metastatic solid tumors in a recent clinical trial, resulted in increased treatment response, by presumably enhancing the dendritic cell cross-talk, and overcoming the immunosuppressive effect of radiation [43]. The above observation is in line with another recent publication in colon cancer, where higher tissue levels of GM-CSF at the tumor cells was correlated with lower cancer stage and prolonged survival [44]. Although the exact mechanism associated with these observed changes remains unknown, based on the presented data, we may safely assume an involvement of the non-canonical Wnt/Ca^2+^ signalling. PFN1 is known to bind to PIP2 and is a key regulator of the balance between cytoskeletal integrity and PIP2 availability for Ca^2+/^PKC signaling [10]. In our study, significant alterations in the expression of phosholipase-C molecules were observed and inactivation/inhibition of Wnt/Ca^2+^ pathway following PFN1 suppression was predicted. Along these lines, alteration of CREB1 and NFkB expression, may explain, in part, the observed differential expression of several genes. At the same time, mRNA changes may also be attributed to transcriptional indirect activity of PFN1 through its interactions with MYPOP transcription factor [45]. These hypotheses warrant further investigation. Even more interestingly, based on our preliminary experiments on a small set of human specimens, a decreased expression trend in all Wnt5b, PLC4b and CREB1 in MIBC (pT2^+^) in comparison to NMIBC (pT1) is suggested. This result is in line with a recent report on CREB1 levels in BC [46]. Even though correlations of PLC4b and Wnt5b to the PFN1 levels per specimen could not be made at significant levels, this preliminary observation supporting similar expression patterns for all three proteins in human tissue, is in line to the presented *in vitro* model predictions. The CREB1 inverse correlation to PFN1 levels per MIBC specimen was unexpected; nevertheless, it may be reflective of activation of alternative mechanisms surpassing the protective effect of PFN1 downregulation in advanced disease. The same may be occurring in the case of angiogenesis where despite the observed decrease in ANGPT1 levels in MIBC in comparison to NMIBC, the tumour is apparently at an increased aggressive state. With no doubt, in depth investigation and extensive experimentation are required to understand the underlying biology of these preliminary observations at the human tissue.Collectively, the present study suggests a role of PFN1 in a well-orchestrated series of events involving cell surface receptors and secreted factors, including CD29, CD49e, and Endothelin-1 (via its interactions with both integrins and PFN1 via which it stabilizes PIP2 phosholipids [47]. Through the latter, an impact on Wnt/Ca^2+^ signaling is suggested with specifically, a decrease in the levels of key members of the pathway (Wnt5b, PLC4b) following PFN1 suppression. Apparently many gaps exist and questions remain unanswered, including delineating the cause(s) and specific role of several of the observed proteomic changes at the secretome. In addition, our study was conducted with one type of BC xenograft model and validation of the main findings was performed based on cross-omics data agreement. Additional experiments involving further BC models, and importantly, analysis of a larger number of human specimens are required to establish applicability of these observations in human disease. Main important questions that we would prioritize include the investigation of a potentially “linker” role of Endothelin-1 in the Integrin - cytoskeletal axis, the links of PFN1 to the anti-tumor cytokine GM-CSF and also a detailed investigation of the correlations of PFN1 to members of the Wnt5/Ca^2+^ signaling pathway (starting by Wnt5b and CREB1) in relation to disease phenotypes.

## MATERIALS AND METHODS

### Ethics statement

The study was designed and performed according to the principles of the Declaration of Helsinki and fullfilling all the requisities of the laws on the protection of patients collaborating in Medical Research. All procedures for the care and treatment of animals were performed according to the institutional guidelines and according to the recommendations of the Federation of European Laboratory Animal Science Associations (FELASA) and approved by the Institutional Animal Care and Use Committee.

### Cell culture and conditioned media collection

T24M cells [48] were cultured in DMEM (Gibco-BRL, Paisley, Scotland, UK) and supplemented with 10% fetal bovine serum (FBS) (Gibco-BRL) at 37°C in 5% CO_2_, as previously described [18, 48]. For the collection of the conditioned media (CM), 1.33x10^3^/cm^2^ cells were cultured in Phenol red-free reduced serum (2%FBS) media (Ltd., Paisley, Scotland) for 24 hours. CM were subsequently collected and centrifuged at 1000 rcf for 10min at 4 °C to remove dead cells and debris. Additional materials and methods regarding the Lentiviral construction are available as supplementary information (Supplementary Text 1).

### Reverse transcriptase (RT) and real time PCR

Total RNA was extracted from cells using the RNeasy Mini Kit (Qiagen Crawley, U.K., http://www1.qiagen.com/). RT–PCR cDNA was synthesized using 1 mg total RNA, M-MLV reverse transcriptase kit (Promega, Madison, WI, USA) and Oligo (dT) primer (Invitrogen, Grand Island, NY, USA) according to the manufacturer's protocol. Quantitative PCR was performed using SYBR Green dye and TaqMan gene expression assays on an Applied Biosystems StepOne Real Time–PCR system (Applied Biosystems, Foster City, CA, USA). Real time PCR was performed using gene-specific primers for PFN1 (Forward: 5′-G C C G G G T G G A A C G C C T A C A T-3' and Reverse: 5′-C CC A G AC G G A G G G C G C G A G T C C −3′) that were mixed with the SYBR Green PCR master mix according to the manufacturer's protocol (Applied Biosystems). Human GAPDH (Forward: 5'-G C A A A T T C C A T G G C A C C G T-3′, Reverse: 5'-T C G C C C C A C T T G A T T T T G G-3′) was used as internal positive control. The reaction was performed in an ABI Prism 7000 apparatus (Applied Biosystems) and the analysis was conducted using the ABI Prism 7700 SDS software (Applied Biosystems). Data were analyzed by the relative quantification (ΔΔ*C*t) method as described previously [49]. The 2^−ΔΔCt^ formula was used to determine fold expression differences. Each assay was performed in duplicates and data represent the mean ± SD of at least two independent experiments.

### Protein extraction and western blot analyses

For protein extraction, cells were collected following trypsinization and cell pellets were re-suspended in isoelectric focusing sample buffer containing 7M Urea (ApliChem Inc., Missouri, USA), 2M Thiurea (Fluka, Sigma Aldrich Co., St. Louis USA), 4% w/v CHAPS (ApliChem Inc., Missouri, USA), 1% w/v DTE, (Sigma Aldrich Co., St. Louis USA) and 2% v/v IPG ampholytes (BioRad Laboratories Inc., CA, USA), followed by bath sonication for 15 minutes [18]. The suspension was centrifuged at 16.000 rcf for 20 min and the supernatants were collected. 3.6% v/v protease inhibitors (Roche) were added to the extracts. Protein concentration was estimated by the use of Bradford reagent (BioRad Laboratories Inc., CA, USA). For western blot analysis, total protein extracts were separated by 15% SDS-PAGE, transferred to Hybond-ECL NC membranes (Amersham Biosciences, Sweden) and probed with the rabbit anti-PFN1 monoclonal antibody at a dilution 1:100 (Santa Cruz Biotechnology, Texas, USA), a mouse monoclonal β-actin antibody at a dilution of 1:2.000 (Santa Cruz Biotechnology, Texas, USA) was used as a control to ensure equal loading. The membranes were subsequently incubated with an anti-rabbit HRP-conjugated secondary antibody (GE Healthcare, United Kingdom) at a dilution 1:10.000 or an anti-mouse HRP-conjugated secondary antibody (Santa Cruz Biotechnology Inc.) at a dilution 1:2.000 and developed using the ECL (Perkin-Elmer, MA, USA) detection system. Films were scanned (GS-800, BioRad scanner) and analyzed using Quantity One software (BioRad Laboratories Inc., CA, USA).

### MTS proliferation assay

T24MshPFN1 or T24MshSCR cells were plated at a density of 10^3^ cells per well in 96-well plates and cultured for 5 days in the presence of DMEM (10% FBS). Media were changed daily, and two different time points (Day 3 and 5) were selected to monitor the proliferation rate. At each time point, CellTiter 96 AQueous One Solution (MTS) (Promega Ltd) was added according to the manufacturer's instructions. The absorbance was recorded at 490 nm with the use of an ELISA plate reader (ELX 800; Biotek Instruments Inc., Winooski, VT). The increase in proliferation was calculated as follows: [(ODdayX-ODday0)/(ODday0)]. Three independent experiments were performed, each including five replicates, and the mean ±SD of each experiment was calculated. Statistical analysis was performed using Student's t test.

### Flow cytometry analysis

T24MshPFN1 and T24MshSCR cells were examined for the expression of CD49e, CD44, CD90, CD29 and CD62P antigens by flow cytometry (Becton Dickinson, San Jose, CA, USA). The appropriate isotype controls were used (Dako, Agilent Technologies) as described previously [48, 49]. F-actin polymerization was tested by FITC-Phalloidin uptake. In brief, after the transduction, cells were incubated with Phalloidin-FITC (Sigma Aldrich Co., St. Louis USA) according to the manufacturer's instructions. Cells were analysed using a Beckman Coulter Cytomics FC 500 flow cytometer (Beckman CoulterLtd, Palo Alto, CA, USA). Apoptosis was determined by Annexin V–FITC staining (BD Biosciences) in T24MshPFN1 or T24MshSCR cells according to the manufacturer's instructions. 7AAD (Sigma Aldrich Co., St. Louis USA) was used for live-dead cell discrimination.

### Transwell migration assay

Transwell migration assays were performed as previously described [18, 50]. Briefly, 4×10^4^ cells T24MshPFN1, T24M or T24MshSCR cells were cultured for 24h in DMEM (2% FBS) and then transferred to a 5μm pore size insert of a transwell plate (Corning-Costar, Cambridge, MA), where they were allowed to migrate for 6h toward CM derived from T24M cells or DMEM (2% FBS) as negative control. As positive control, recombinant PFN1 protein (Abcam plc, Cambridge, MA, USA) (1μg/ml) was added in DMEM (2% FBS). Two independent experiments were performed, each including two replicates, and the mean of each experiment was calculated. Statistical analysis was performed using Student's t test.

### *In vitro* colony formation (CFU) assay

Ten thousand T24MshPFN1 or T24MshSCR cells were cultured in DMEM (10% FBS), containing 5 mg/ml of Matrigel (BD, New Jersey, USA). Cells were cultured on coverslips in 24-well plates in the matrigel-containing media and subsequently placed in a 5% CO_2_ incubator at 37°C for 1hour. DMEM (10% FBS) was then added on the top of the gel. Cells were left to grow in a 3D culture for 12 days and the media were changed every 2 days. The CFU formation was monitored weekly. Three independent experiments were performed, each including two replicates, and the mean±SD of each experiment was calculated. Statistical analysis was performed using Student's t test.

### Adhesion assay

Ten thousand T24MshPFN1 or T24MshSCR cells were plated in a 96-well vessel treated with fibronectin (10μg/ml) (Sigma-Aldrich). Cells were incubated in DMEM (10% FBS) at 37°C in 5% CO_2_ for 10, 20 and 30 minutes, respectively. The non-adherent cells were removed by washing with PBS (Lonza Group Ltd.). The adherent cells were then fixed using 4% paraformaldehyde (Sigma-Aldrich) and stained with crystal violet (Sigma Aldrich Co.). Adhesion was quantified by measuring the absorbance at 595 nm. Images from the stained nuclei were taken using a Leica DMLS2 Microscope (Leica Microsystems IR GmbH). Three replicates were used per condition. Statistical analysis was performed using Student's t test.

### Proteome profiler array of conditioned media (CM)

For the preparation of the CM, 1×10^6^ T24MshPFN1 or T24MshSCR cells were cultured until 80% confluent, and the media were replaced with DMEM containing 0.5% (v/v) FBS to prevent protein aggregation. The cells were cultured for a further 24 hours and the CM was collected and concentrated approximately 10-fold using ultra filtration units with a 3-kDa cut-off (Millipore Ltd. Ltd.), and analyzed for specific proteins using proteome profiler arrays for angiogenesis growth factors (Catalog #ARY007, R&D Systems Inc., Minneapolis, USA) according to the manufacturer's instructions. Quantitation of the detected spots was performed using the Quantity One Software 4.4.1 (BioRad Laboratories Inc., Amersham, England). Two replicates per sample were analyzed. Results for the different growth factors assayed are expressed as means±SD and statistical analysis was performed using Student's t test.

### Animal experiments

NOD-SCID mice were purchased from the Jackson Laboratory (JAX Mice & Services Bar Harbor, ME USA) and housed and maintained at the Animal Facility of the BRFAA. For tumor formation, 3.3×10^6^ T24MshPFN1 cells (n=13), T24MshSCR (n=19) or T24M untransduced cells (n=16) were administered subcutaneously (s.c.) into the tail base of 6 to 8-week old male NOD/SCID mice of 22-24gr weight. Tumor size was measured by caliper weekly for at least 3 months or until the presence of a tumor diameter >17mm, tumor ulceration or bleeding, when in those cases mice were sacrificed earlier. For examining tumor growth and/or detecting metastasis by bioluminescence imaging, mice were injected with luc-T24MshPFN1 and luc-T24MshSCR cells. Mice were initially injected with luciferin (1 mg/ml, Promega Corporation). Mice were then anesthetized using Ketamine HCl, xylazine, NaCl, 0.9% (GE Heathcare, UK). The *in vivo* bioluminescense monitoring was performed in a Xenogen IVIS Lumina II System (Advanced Molecular Vision, Inc.). Animals were sacrificed and lung, spleen, kidneys and liver were collected for monitoring metastasis.

### Immunohistochemistry in murine tumor specimens

Tissues from mouse tumors or organs were fixed in 10% formalin (Sigma-Aldrich) and 5μm thick paraffin sections were prepared, as previously described [18]. Tissue sections were dewaxed in xylene (Carlo-Erba Reagents, Milano, Italy) and then rehydrated in graded alcohol (Sigma-Aldrich). Endogenous peroxidase activity was blocked with 0.3% (v/v) hydrogen peroxide (Sigma-Aldrich) in methanol (AppliChem GmbH, Darmstadt, Germany). Non-specific binding was blocked using 10% (v/v) fetal bovine serum (Gibco-BRL) in PBS. Sections were subsequently incubated with rabbit anti-Profilin1 monoclonal antibody (Enzo, Life Sciences, NY, USA), or appropriate isotype controls. The reaction was developed with biotinylated goat anti-rabbit secondary antibody (DakoCytomation Ltd, Cambridgeshire, UK), followed by ABC-complex-HRP (DakoCytomation) and DAB (Vector Laboratories, Burlingame, CA, USA) reagents. Finally, the sections were counterstained in Gill's hematoxylin (Sigma-Aldrich). To evaluate the staining for PFN1 expression in murine tissue, an image analysis approach was implemented by Image J software 1.38× software (http://rsb.info.nih.gov/ij/). For CD31 stainings a rabbit anti-CD31 polyclonal antibody (Abcam, ab28364) was used in dilution of 1:250. Quantitation of signal was assessed by Image J software 1.38× software (http://rsb.info.nih.gov/ij/).

### Immunohistochemistry in human BC specimens

Tissues specimens from a total number of 24 patients [21-male, 3-female] with BC were used for IHC following approval of the study by the local Ethics Committee (Laikon Hospital: ES76). Tumor staging was classified according to TNM/UICC (2009) classification as follows: 9 pTa cases [mean age: 63±12], 8 pT1 cases [mean age: 70±9] and 7 T2^+^ cases [mean age: 64±7] (Supplementary Table S1). In situ carcinoma co-existed in 6 cases, concomitantly to 4 pT2^+^ and 2 pT1 bladder cancer cases (Supplementary Table S1). Immunohistochemical staining for PFN1 was performed as previously described [51]. In brief, endogenous peroxidase activity was quenched with 0.3% hydrogen peroxide in Tris-buffered saline (TBS). After rinsing with TBS, normal horse serum was applied to block non-specific antibody binding. Subsequently, sections were incubated overnight at 4°C with rabbit anti-Profilin1 monoclonal antibody (Enzo, Life Sciences, NY, USA), in 1:500 dilution or appropriate isotype controls. A three-step procedure (Avidin-Biotin-Peroxidase, Vector Laboratories, Burlingame, CA, USA) was used for visualization, with diaminobenzidine as a chromogen according to the kit instructions. Finally, sections were counterstained with hematoxylin and mounted. A quantitative approach was then followed to confirm pathologists' observations using the Image J software (http://rsb.info.nih.gov/ij/) [52]. In brief, tissue sections were scanned under the microscope using X20 magnification. Depending on tissue size, different number of images (5-10) were acquired per section. Ten-50 fields of identical area dimensions per image were selected for mesurement analysis. Optical density was normalized over the unstained tissue substrate and mean intensity values were estimated, as previously described [51]. Mean values of intensity were calculated per image and subsequently for each section. All raw data-measurements per sample are provided in Supplementary Table S1. Similarly, for PLCb4 staining a rabbit anti-PLCb4 monoclonal antibody (SANTA CRUZ BIOTECHNOLOGY, INC sc-166131) was used in 1:50 dilution, for Wnt5b (Abnova H00081029-B01P) in 1:50 dilution, for CREB1 (CSB-PA005947HA01HU) in 1:50 dilution and for ACVR1C (CSB-PA854112ESR1HU) in dilution 1:100. As a secondary antibody the Envision FLEX/HRP DAKO K8023 (part of kit) was employed. As previously, the tissue sections were scanned under the microscope using X40 magnification. Optical density was normalized over the unstained tissue and mean intensity values were estimated, as previously described [51]. Mean values of intensity were calculated per image and subsequently for each section. All raw data-measurements per sample are provided in Supplementary Table S1.

The statistical analysis was based on two-tailed Student's T-test of unequal variance. Values of *p*<0.05 were considered as statistically significant. Correlation analysis of the tissue staining measurements at the human BC patients level was performed using Spearman rank correlation coefficient. Correlation was marked as significant for *p*<0.05. The correlation analysis was performed with SPSS software v.22 (SPSS Inc, USA).

### Total mRNA sequencing

Total RNA was isolated from T24MshPFN1, T24MshSCR or T24M cells using the RNeasy kit (Qiagen, Valencia, CA, USA), as described previously and according to the manufacturer's protocol. Concentration and purity of RNA were assessed using the NanoDrop ND 1000 spectrophotometer (Nanodrop products, Wilmington, DE, USA) at 260 nm. In total 6 samples were analyzed including T24MshPFN1, T24MshSCR or T24M cells (in two biological replicates each). The preparation of libraries and the sequencing of the mRNA along with the analysis of the raw data were performed by GenomeScan B.V Company. The RNA concentration was assessed using the Life Technologies Qubit (Thermo Fischer Scientific, Waltham, MA USA). Further evaluation of the quality and integrity of isolated RNA was conducted using Agilent Bioanalyzer (Agilent Technologies Inc., Santa Clara, USA). Subsequently, samples were processed by Illumina^®^ mRNA-Seq Sample Prep Kit (Illumina, Inc, San Diego, USA) according to Illumina protocol. Briefly, mRNA isolation was performed using oligo-dT magnetic beads (Illumina, Inc) followed by the mRNA fragmentation and cDNA synthesis. For the latter, the quality and yield was measured with Lab-on-a-Chip analysis and confirmed the expected size of the product in the range between 200-500 bp. Clustering and DNA sequencing was performed using Illumina cBot (Illumina, Inc) and HiSeq2500 (Illumina, Inc), in line with manufacturer's instructions at the concentration of 16pM of DNA. Image analysis, base calling and quality check was conducted with the Illumina data analysis pipeline RTAv1.18.64 (Illumina, Inc) and Bclfastqv1.8.4 (Illumina, Inc). Data obtained from the HiSeq2500 in fastq format was used as source for the downstream data analysis. Alignment of fastq reads was performed using TopHat version 2.0.12 [53] (Johns Hopkins University) against the assembled human genome GRCh37.p13 with the corresponding Ensembl release 75 annotation [54] (http://grch37.ensembl.org/index.html). The alignment run with default parameters but allowing for a genome multihit search and transcriptome build and mapping. Alignment quality metrics were collected using Qualimap version 2.0.1 [55] (Max Planck Institute For Infection Biology and Bioinformatics Department). Quantification of feature alignments was performed using HTSeq-counts from HTSeq framework version 0.6.1p1 [56] (Genome Biology Unit, EMBL Heidelberg). Default parameters were used for a non-stranded RNA-seq library using the intersection non empty algorithm to count reads that fall into annotated gene locus. Finally, the normalization of the count data and statistical analysis for the differential expression was performed with DESeq2 package version 1.6.3 [57] for R statistical computing software [15].

### Functional annotation and in silico analysis

*In silico* pathway analysis was performed in order to predict significantly altered pathways upon downregulation of PFN1. The differentially expressed transcripts were assigned to their corresponding proteins and functionally annotated using Gene Ontology annotations retrieved from Uniprot-GOA annotations [58] and/ or NeXtProt database [59]. They were also mapped to pathways using QIAGEN's Ingenuity® Pathway Analysis software (IPA®, QIAGEN Redwood City, www.qiagen.com/ingenuity). Statistical analysis was conducted by using right-tailed Fisher's exact test and pathways with a *p* value below 0.05 were considered as significant.

## SUPPLEMENTARY DATA









## Abbreviations

ACVN1: Activin A
ADP: Adenosine diphosphate
ANGPT1: Angiopoietin 1
ANGPT2: Angiopoietin 2
ATP: Adenosine triphosphate
au: arbitrary units
BC: Bladder Cancer
CAMKIV: Calcium/calmodulin-dependent protein kinase type IV
CCL2: Chemokine (C-C Motif) Ligand 2
CD31: cluster of differentiation 31
CFU: colony-forming unit
CREB: cAMP response element-binding protein
CXCR4: C-X-C chemokine receptor type 4
CXCL12: C-X-C motif chemokine 12
DPPIV: Dipeptidyl peptidase 4
EDN-1: Endothelin-1
FACS: fluorescence-based flow cytometry
FGF-1: Fibroblast growth factor-1
FITC: fluorescein isothiocyanate
GM-CSF: Granulocyte-macrophage colony-stimulating factor
IHC: Immunohistochemistry
MFI: Median Fluorescence Intensity
NFkB: nuclear factor 'kappa-light-chain-enhancer' of activated B-cells
NOD/SCID: non-obese diabetic severe combined immunodeficient mouse
OD: Optical Density
PECAM-1: Platelet endothelial cell adhesion molecule
PEDF: Pigment epithelium-derived factor
PFN1: Profilin-1
PLG: Plasminogen
PLCb2: 1-phosphatidylinositol 4; 5-bisphosphate phosphodiesterase beta 2
PLCb4: 1-phosphatidylinositol 4; 5-bisphosphate phosphodiesterase beta 4
PIP2: Phosphatidylinositol 4,5-bisphosphate
PIP3: Phosphatidylinositol (3,4,5)-trisphosphate
TNFSF15: Tumor necrosis factor ligand superfamily member-15
TRIM31: Tripartite motif containing 31
TYMP: Thymidine phosphorylase
VLA-5 antigen: very late antigen-5 or Integrin alpha5, beta1 (CD29/CD49e).
