# Supplementary material for: Silencing of Profilin-1 suppresses cell adhesion and tumor growth via predicted alterations in integrin and Ca2+ signaling in T24M-based bladder cancer models
Source: Oncotarget. 2016 Sep 23;7(43):70750–68. doi: 10.18632/oncotarget.12218 (PMC5342587; doi:10.18632/oncotarget.12218)
Supplement: Supplementary file 1 [file oncotarget-07-70750-s001.pdf]

Silencing of Profilin-1 suppresses cell adhesion and tumor growth via predicted alterations in integrin and Ca^{2+} signaling in T24M-based bladder cancer models

SUPPLEMENTARY DATA

Supplementary Text 1

Additional materials and methods and detailed protocols for the total mRNA sequencing analysis, immunohistochemistry analysis and additional in vitro experiments.

Lentiviral vectors and cell transduction

A four-plasmid lentiviral expression system was employed for the generation of shPFN1 lentivirus. In brief, pMD2.VSVG, pMDLgag/pol/RRE and pRSV-REV plasmids were transiently co-transfected with pLKO.1 WPRE vector (Sigma Aldrich Co., St. Louis USA) that includes shRNA sequence complementary to PFN1 (shPFN1) or shRNA non targeting, scrambled sequence (shSCR) under the human Phospho-glycerate Kinase promoter (PGK) in HEK293T cells. The produced viruses were collected and concentrated by using Amicon Ultra Centrifugal Filters-100K Units (Merck KGaA, Darmstadt, Germany), as described previously [1]. Two different viruses were produced: a) the shPFN1 virus, for stable knockdown of Profilin-1, b) shSCR, as a scrambled plasmid control virus. For the lenti-viral production concerning the Luciferase gene, the pCCLsin.PPT.hPGK.WPRE-luc expression vector was employed. The shPFN1 and shSCR lentiviral titers were determined by transduction of HT1080 cells using serial dilutions of the concentrated viral stocks. Colonies were stained with Crystal violet (Sigma Aldrich Co., St. Louis USA) and counted after 15 days. The titers ranged from $1.35x10^7$ to $1.15\ x10^9$ for shPFN1 and $1.57\ x10^6$ to $1.3x10^8$ (TU/ml) respectively. T24M cells were transduced with shPFN1 or shSCR lenti-viruses at a multiplicity of infection (MOI) of 30 and 2 days after puromycin selection (0.6μg/ml) was employed. The titer for the virus containing the Luciferase transgene was determined by Real Time PCR using specific primers for the wpre gene (5′-T T C T C C T C C T T G T A T A A A T C C T G G T T -3′ and Reverse: 5′- C G C C A C G T T G C C T G A C A -3′). To estimate the number of the vector copy number, a standard curve was generated, based on the GFP vector copy numbers determined by Real time PCR and flow cytometry analysis analyses (y = -0.3262x-1.3784), as previously described [2]. The luciferase viral titers ranged from $1.27x10^6$ to $3.87x10^8$ TU/ml and MOI of 100 was used for LUC viral transduction.

Cloning of the reporter gene luciferase (luc+) plasmid vector in PCCLsin.PPT.hPGK.eGFP.WPRE

The luciferase gene originally expressed in the firefly (Photinus pyralis) is widely used in the labeling of eukaryotic cells. In this experiment, the open reading frame of the firefly luciferase gene (luc+) was amplified in the plasmid vector pGL3-Basic Vector (Promega), using polymerase chain reaction (PCR). The primers employed to amplify the luciferase gene, were designed as follows:

Forward Primer: 5′CTGAGATCG/GATCCAAGCCACCATGGAAGACGCC-3′

Restriction enzyme: BamHI

Reverse Primer: 5′GATCGTCGA/CCTTACACGGCGATCTTTCCGCCCTT-3′

Restriction enzyme: SalI

The luciferase gene (luc +) was subsequently cloned into the GFP (encoding for Green Fluorescent Protein) plasmid vector pCCLsin.PPT.hPGK.eGFP.WPRE (kindly provided by Prof Luigi Naldini, The San Raffaele Telethon Institute for Gene Therapy, Milan). For the cloning process, the PCR product as well as the pCCLsin.PPT.hPGK.eGFP.WPRE plasmid vector were initially digested with BamHI and SalI restriction enzymes and ligated accordingly. The same vector was further utilized for the generation of a LUC lentivirus.

REFERENCE

1. Trohatou, O., D. Zagoura, V. Bitsika, K.I. Pappa, A. Antsaklis, N.P. Anagnou, and M.G. Roubelakis, Sox2 suppression by miR-21 governs human mesenchymal stem cell properties. Stem Cells Transl Med, 2014. 3: p. 54-68.

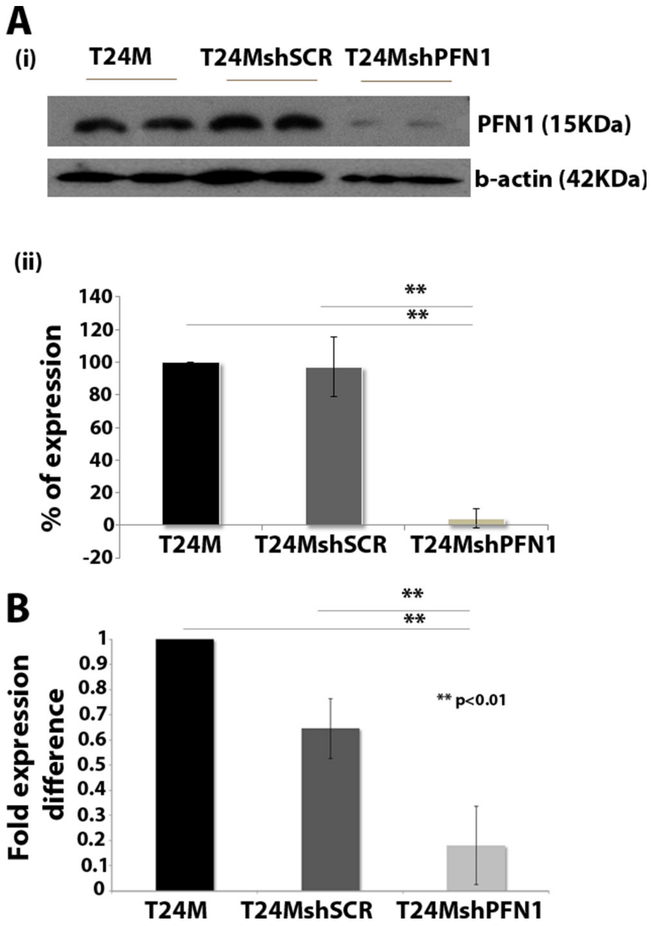

Supplementary Figure S1: Representative image of western blot for PFN1 expression in cell extracts of T24M, T24MshSCR and T24MshPFN1 cells. (ii) Quantitative analysis of PFN1 expression in T24MshSCR and T24MshPFN1 cells, normalized to the PFN1 expression levels of T24M cells. Quantification was performed by using Quantity One software and the results were normalized to the b-actin positive control. Values are means ± S.D. for four independent experiments (**$p < 0.01$, Student's t-test). **B.** Representative plot of mRNA fold expression levels of PFN1 in T24M, T24MshSCR and T24MshPFN1 cells. Values are means ± S.D. for four independent experiments, (**$p < 0.01$, Student's t-test).

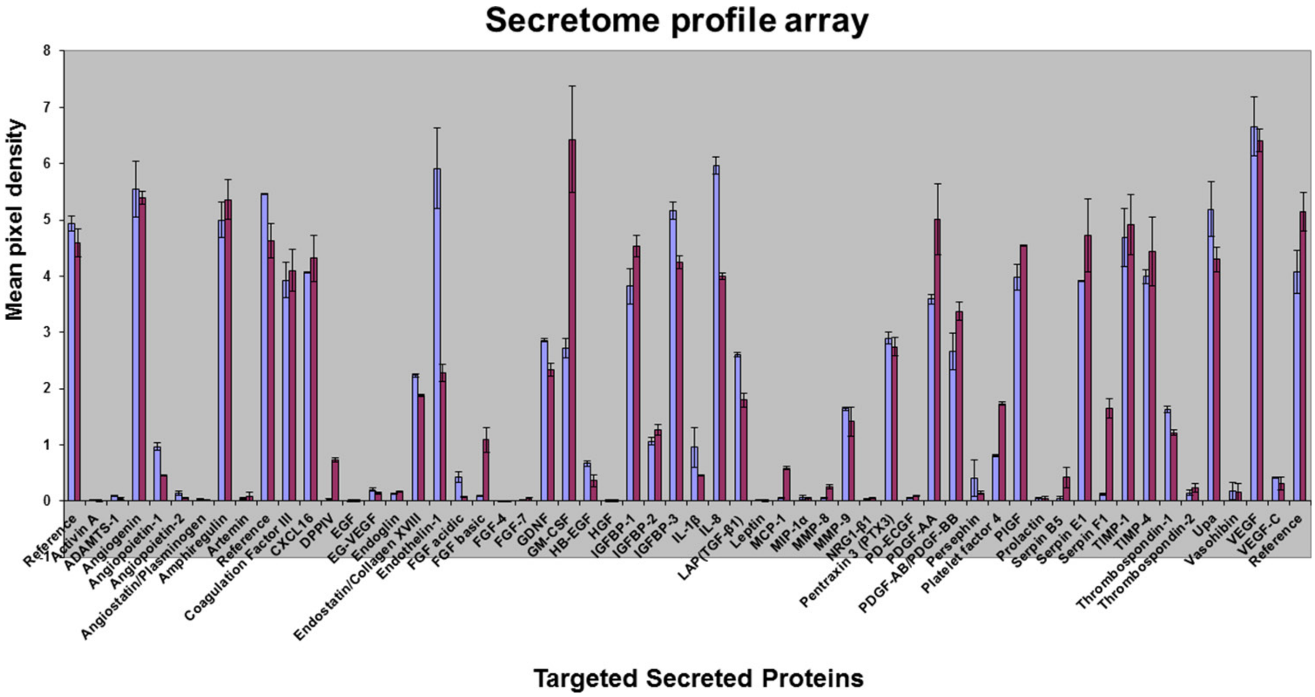

Supplementary Figure S2: Analysis of angiogenic factors secreted by T24MshPFN1 and T24MshSCR cells. Quantification of selected secreted factors was performed using Quantity One image software. The quantification of the secreted levels are presented in bar graphs for for T24MshPFN1 and T24MshSCR cells.

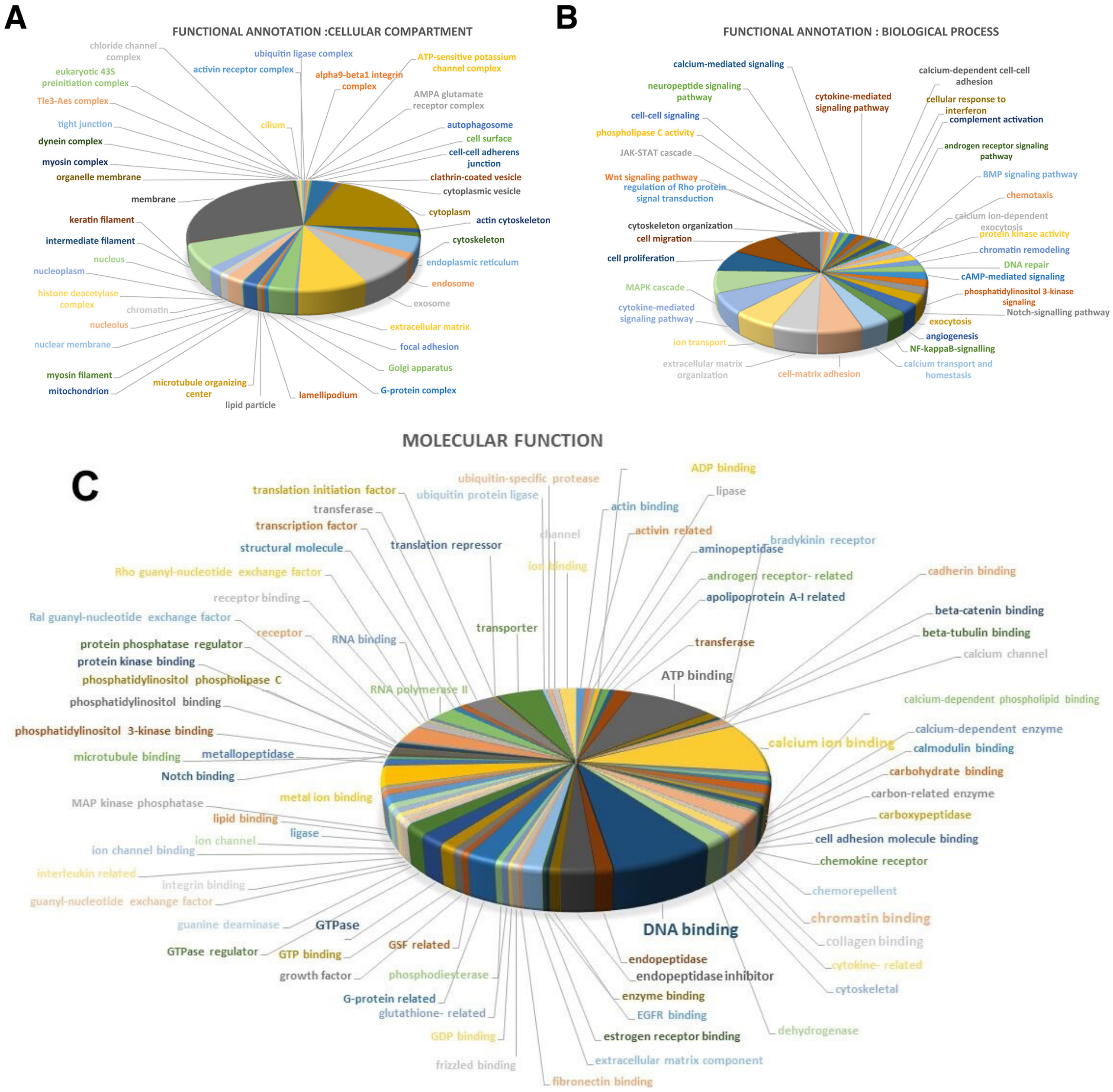

Supplementary Figure S3: Functional annotation of the 652 differentially expressed protein encoding genes based on manual interpretation of the GO data, derived from the Uniprot database. Distribution according to **A.** subcellular localization. **B.** biological process and **C.** Molecular Function.

Supplementary Table S1: Clinical and demographical data for bladder cancer tissue specimens that were employed for Immunohistochemical analysis to investigate PFN1 expression at the tissue level (Spreadsheet 1). Quantification of the signal intensity was performed using ImageJ software. The normalised values of the stained over the unstained area are provided for each section. Quantification of the optical absorbance of the Immunohistochemical analysis for PFN1 tissue staining (Spreadsheet 2), PLCb4 (Spreadsheet 3), Wnt5b (Spreadsheet 4), CREB1 (Spreadsheet 5) and AGPT1 (Spreadsehhet 6) is reported.

See Supplementary File 1

Supplementary Table S2: RNA sequencing data from the analysis of T24MshPFN1. T24MscPFN1 and T24M cells (Illumina Platform) Spreadsheet 1: List of the 654 protein encoding differentially expressed genes that were identified during the total RNA sequencing analysis. The most prominent differentially expressed genes were defined accounting for fold change magnitude of 1.5. Spreadsheet 2: List of 44 pathways, as revealed by the IPA pathway mapping analysis. With red font those pathways directly associated with PFN1.

See Supplementary File 2

Supplementary Table S3: Data integration including transcriptomics data, antibody- based analysis of secreted factors and cell–surface receptor analysis. Agreement between the datasets is investigated in terms of significant alterations between the shortlisted targets pointing towards the same regulation of expression.

See Supplementary File 3
